# Advanced MRI in neuro-oncology: can we proceed without inclusion of energy metabolism?

**DOI:** 10.18632/oncotarget.27015

**Published:** 2019-06-18

**Authors:** Andreas Stadlbauer, Michael Buchfelder, Arnd Dörfler, Stefan Oberndorfer

**Affiliations:** Institute of Medical Radiology, University Clinic of St. Pölten, St. Pölten, Austria; Department of Neurosurgery, University of Erlangen-Nürnberg, Erlangen, Germany

**Keywords:** glioblastoma, magnetic resonance imaging, intratumoral heterogeneity, tumor microenvironment, Warburg effect

Altered energy metabolism is recognized as a core hallmark of cancer. Cancer cells can reprogram their metabolism to promote cellular growth and proliferation, adapt nutrient or oxygen depleted environments, and escape immune surveillance. A common phenotype is an increased glucose and glutamine uptake, which is metabolized through glycolysis into lactate regardless of oxygen availability. Known as aerobic glycolysis or Warburg effect [1], this pathway provides metabolic intermediates critical for numerous biosynthetic processes, conferring proliferative advantages, and acidifies the tumor microenvironment. Based on current research, however, a new hypothesis is developed, the reverse Warburg effect [2]: Cancer cell production of reactive oxygen species (ROS) inactivates caveolin 1 in adjacent stromal fibroblasts. This induces mitophagy, reduces mitochondrial function, and increases lactate production via glycolysis in these cancer-associated fibroblasts. Lactate secreted by stromal cell fuels cancer cells’ oxidative metabolism in mitochondria, which drives tumor growth and proliferation. This complexity of metabolic interactions is in accordance with the well-known intratumoral heterogeneity of genetic and phenotypic alterations. Therefore, detailed analysis and imaging of energy metabolism may describe how cancer cells respond to environmental changes or drug treatment.

Of all cancers, glioma is responsible for the highest number of lost life years [3]. Despite standard therapy, which includes maximum save and radical resection, concomitant radiochemotherapy, and adjuvant chemotherapy, median survival of glioblastoma patients (comprising 80% of glioma cases) is still only 12–16 months [4]. Reprogramming of energy metabolism and diversity of metabolic phenotypes have been associated with treatment failure and tumor progression [5]. Furthermore, even advanced MRI approaches (e.g. MR perfusion) and improved criteria, such as those provided by the Response Assessment in Neuro-Oncology (RANO) group (6), are limited to reliably detect treatment response as well as to distinguish active tumor tissue and tumor progression from pseudoprogression, reactive tissue, and pseudoresponse, respectively. Especially glioblastoma comprises several metabolic subtypes, each with distinguishing hallmark mutations and clinical features [7]. The underlying metabolic differences between these subtypes are largely unanswered. Detecting and addressing subgroup-specific metabolic requirements may here lead to a more personalized imaging and may also become of interest for future treatment planning.

The number of methods for noninvasive *in-vivo* assessment of energy metabolism, however, is extremely limited. The key metabolites for the pathways of energy metabolism are glucose, oxygen, glutamine, and lactate. The gold-standard for clinical imaging of glucose metabolism is positron emission tomography (PET), which requires the radiolabeled glucose analog ^18^F-fluoro-D-glucose (^18^F-FDG). FDG, like normal glucose, enters the cell via glucose transporters and is phosphorylated by hexokinase to form ^18^F-FDG-6-phosphate. The 2-hydroxyl group (-OH) of normal glucose is needed for further glycolysis to pyruvate, but ^18^F-FDG-6-phosphate is missing this 2-hydroxyl – the ^18^F atom is at this position. This intermediate is unable to be further catabolized and becomes trapped in the cell. PET tracers for the other key metabolites (^18^F-fluoroglutamine, ^11^C-glutamine, ^11^C-lactate, ^15^O) are available but have limited clinical applicability. Hence, PET has several drawbacks with respect to imaging of metabolic pathways: (**i**) the use of radioactive agents may limit the number of exams; (**ii**) imaging of the key metabolites of energy metabolism requires injection of multiple tracers; (**iii**) ^18^F-FDG becomes trapped in the cell and inhibits the pathway.

MRI has unparalleled soft tissue contrast resolution compared with other imaging modalities. Less appreciated is the potential of MRI to study energy metabolism. The sensitivity of MRI to changes in hemoglobin oxygenation through the blood oxygen level-dependent (BOLD) effect, which is the basis of functional MRI (fMRI), enables the measurement of baseline brain tissue oxygenation via quantification of the BOLD signal. Additionally, there exists a strong relationship between energy metabolism pathways, oxygen and nutrient supply, and tumor neovasculature. MRI is the modality of choice for the non-invasive examination of tumor vasculature.

Quantitative BOLD (qBOLD) and vascular architecture mapping (VAM) were the basis for our previous study [8], where we fused MRI biomarker information for oxygen metabolism and neovascularization using an automatic classification algorithm for assessment of the dominating metabolic strategy for energy production within the heterogeneously structured tumor microenvironment (TME). Our approach, which we termed “TME mapping”, uncovered two different metabolic phenotypes for newly diagnosed glioblastoma IDH1 wildtype: (i) A glycolytic dominant phenotype with stable functional neovasculature; and (ii) a necrotic/hypoxic dominant phenotype with high proportion of unstable defective dysfunctional neovasculature and a more aggressive tumor behavior. The glycolytic phenotype showed longer progression-free survival. However, our approach was only one step on the way to MRI of energy metabolism – or Warburg MRI.

The MRI modality called chemical exchange saturation transfer (CEST) opens new opportunities for mapping glucose metabolism by using natural glucose as a MRI contrast agent [9]. Proton MR spectroscopic imaging (^1^H-MRSI), on the other hand, is a metabolic MRI modality for quantification of metabolites primarily associated with energy metabolism such as glutamine and lactate [10], but not glucose. The feasibility of dynamic glucoCEST and ^1^H-MRSI for clinical applications was demonstrated in glioma patients.

So, multiple pieces of the puzzle are on the table. Interdisciplinary cooperation including physicians, biologists and physicists is required to put the pieces together. This may allow us to refocus the MRI lens towards energy metabolism for better *in vivo* understanding of tumor biology and may enable us for the future targeting glioma in a more personalized approach. Visualizing energy metabolism of glioma by MRI is still far away from routine use, but in our opinion has great potential for individualized tumor characterization and possibly opens doors for future tailored treatment approaches.
